# Net clinical benefit of dabigatran vs. warfarin in venous thromboembolism: analyses from RE-COVER^®^, RE-COVER™ II, and RE-MEDY™

**DOI:** 10.1007/s11239-017-1479-z

**Published:** 2017-02-16

**Authors:** Martin Feuring, Sam Schulman, Henry Eriksson, Ajay J. Kakkar, Sebastian Schellong, Stefan Hantel, Elke Schueler, Jörg Kreuzer, Samuel Z. Goldhaber

**Affiliations:** 1 Boehringer Ingelheim GmbH & Co. KG, 55216 Ingelheim am Rhein, Germany; 2 Department of Medicine, McMaster University and Thrombosis and Atherosclerosis Research Institute, Hamilton, ON Canada; 3 Department of Medicine, Sahlgrenska University Hospital-Östra, Gothenburg, Sweden; 4 Thrombosis Research Institute and University College London, London, UK; 5 Medical Division 2, Municipal Hospital Dresden-Friedrichstadt, Dresden, Germany; 6 Accovion GmbH, Eschborn, Germany; 7 Cardiovascular Division, Brigham and Women’s Hospital, Harvard Medical School, Boston, MA USA

**Keywords:** Venous thromboembolism, Warfarin, Dabigatran etexilate, Anticoagulant

## Abstract

The direct oral anticoagulants, e.g., dabigatran etexilate (DE), are effective and well tolerated treatments for venous thromboembolism (VTE). Net clinical benefit (NCB) is a useful concept in weighing potential benefits against potential harm of comparator drugs. The NCB of DE vs. warfarin in VTE treatment was compared. Post-hoc analyses were performed on pooled data from the 6-month RE-COVER® and RE-COVER™ II trials, and data from the RE-MEDY™ trial (up to 36 months), to compare the NCB of DE (150 mg twice daily) and warfarin [target international normalized ratio (INR) 2.0–3.0]. Patients (≥18 years old) had symptomatic proximal deep vein thrombosis and/or pulmonary embolism. NCB was the composite of cardiovascular endpoints (non-fatal events of recurrent VTE, myocardial infarction, stroke or systemic embolism), all-cause death, and bleeding outcomes, all weighted equally. A broad definition of NCB included major bleeding events (MBE) and clinically relevant non-major bleeding events as bleeding outcomes, while a narrow definition included just MBE. The pooled dataset totalled 5107 patients from RE-COVER/RE-COVER II and 2856 patients from RE-MEDY. When NCB was narrowly defined, NCB was similar between DE and warfarin. When broadly defined, NCB was superior with DE vs. warfarin [RE-COVER/RE-COVER II, hazard ratio (HR) 0.80; 95% confidence interval (CI), 0.68–0.95 and RE-MEDY, HR 0.73; 95% CI 0.59–0.91]. These findings were unaffected by warfarin time in therapeutic range. The NCB of DE was similar or superior to warfarin, depending on the NCB definition used, regardless of the quality of INR control.

## Introduction

The direct oral anticoagulants (DOACs) are effective and usually well tolerated for treating venous thromboembolism (VTE) [1–7]. In pooled analyses from the RE-COVER^®^ and RE-COVER™ II trials in patients with acute VTE, dabigatran etexilate (DE) at a fixed dose of 150 mg twice daily was as effective as warfarin [dose adjusted to achieve international normalized ratio (INR) between 2 and 3] for the treatment of acute VTE. For prevention of recurrent VTE, DE was associated with a significantly lower risk of clinically relevant, major or non-major, bleeding and of any bleeding events [6]. In the RE-MEDY™ trial of extended anticoagulation, DE was non-inferior to warfarin for the prevention of recurrent VTE, with a significantly lower risk of major or clinically relevant non-major bleeding [8].

The benefit–risk balance of DE compared with warfarin in VTE treatment and prevention of recurrence can be further understood by assessing the net clinical benefit (NCB) [9]. NCB weighs potential benefits (e.g., reduced risk of VTE or stroke) vs. potential harm (e.g., risk of bleeding). Thus, NCB quantifies both clinical efficacy and safety outcomes. NCB is particularly useful in the assessment of multiple endpoints affecting mortality and morbidity [e.g., VTE, myocardial infarction (MI), stroke, major bleeding events (MBEs), clinically relevant non-major bleeding events (CRNMBEs)] and for facilitating the comparison of the benefit–risk balance of anticoagulants.

The effectiveness and safety of warfarin depends on the time in therapeutic range (TTR) with an INR between 2.0 and 3.0 [10]. Analysis of the NCB of dabigatran compared with that of warfarin at high TTRs will determine whether the comparative NCB is affected when INR is tightly controlled.

Post-hoc analyses were performed on pooled data from RE-COVER and RE-COVER II, as well as data from RE-MEDY, to compare the NCB of DE with that of warfarin overall, and in relation to mean TTR for warfarin at each center (cTTR). Broad and narrow definitions of NCB were used: MBEs plus CRNMBEs as bleeding outcomes and MBEs as the only bleeding outcome, respectively.

## Methods

### Study population and trial design

The study designs, populations and outcomes of the RE-COVER, RE-COVER II, and RE-MEDY trials have been published [5, 6, 8]. In all three trials, patients aged ≥18 years with objectively confirmed symptomatic proximal deep vein thrombosis or pulmonary embolism were eligible for inclusion.

In RE-COVER and RE-COVER II, patients were randomized to warfarin or warfarin–placebo plus parenteral anticoagulation for ≥5 days until the INR was ≥2 on two consecutive measurements. Parenteral therapy was then discontinued and patients continued warfarin (therapeutic INR range 2.0–3.0) or received DE 150 mg twice daily for 6 months (double-dummy treatment period).

In RE-MEDY, patients who had been treated for 3–12 months with an approved anticoagulant (or were participating in RE-COVER or RE-COVER II) were randomized to DE 150 mg twice daily or warfarin (INR range 2.0–3.0) for 6–36 months.

### Study outcomes

For this post-hoc analysis, NCB was evaluated as the composite of cardiovascular endpoints (the components being non-fatal events of recurrent VTE, MI, stroke or systemic embolism), all-cause death, and bleeding outcomes, which were all weighted equally. The bleeding outcomes either included MBEs alone (narrow definition of NCB) or MBEs plus CRNMBEs (broad definition of NCB). MBEs and CRNMBEs were defined according to the International Society on Thrombosis and Haemostasis criteria (MBEs) [11], and as previously defined for the phase 3 dabigatran studies (CRNMBEs) [6]. All events were evaluated from the beginning of the parenteral phase of anticoagulation treatment until the end of the post-treatment period (RE-COVER and RE-COVER II) or from randomization to the end of the planned treatment period (RE-MEDY).

### Statistical analyses

Outcomes were analyzed with a Cox proportional hazards model. Statistical analyses were performed with SAS^®^ version 9.2 (Cary, NC, USA).

## Results

### Population

The pooled dataset from RE-COVER and RE-COVER II included 2553 patients randomized to DE and 2554 patients randomized to warfarin [6]. The RE-MEDY dataset consisted of 1430 and 1426 patients randomized to DE and warfarin, respectively [8]. Patient characteristics were generally similar between DE and warfarin groups in the pooled RE-COVER/RE-COVER II dataset and in RE-MEDY (Table 1).

**Table 1:** Characteristics of patients receiving dabigatran or warfarin in RE-COVER/RE-COVER II pooled data and RE-MEDY

	Including CRNMBE^a^		Excluding CRNMBE^a^	
	Dabigatran (*n* = 2553)	Warfarin (*n* = 2554)	Dabigatran (*n* = 1430)	Warfarin (*n* = 1426)
Age, years, mean ± SD	54.8 ± 16.0	54.7 ± 16.2	55.4 ± 15.0	53.9 ± 15.3
Female sex, n (%)	1033 (40.5)	1033 (40.4)	559 (39.1)	555 (38.9)
Race or ethnic group, n (%)				
White	2206 (86.4)	2193 (85.9)	1288 (90.1)	1284 (90.0)
Black	54 (2.1)	51 (2.0)	29 (2.0)	28 (2.0)
Asian	292 (11.4)	310 (12.1)	113 (7.9)	114 (8.0)
Missing	1 (0.0)	0 (0.0)		
Weight, kg, mean ± SD	84.3 ± 19.4	83.6 ± 19.0	86.1 ± 19.3	86.0 ± 18.9
Estimated creatinine clearance, ml/min, mean ± SD	107.0 ± 42.2	105.8 ± 40.5	104.2 ± 38.6	106.6 ± 37.9
Type of qualifying event^b^, n (%)				
DVT only	1755 (68.7)	1744 (68.3)	938 (65.6)	922 (64.7)
PE only	569 (22.3)	567 (22.2)	324 (22.7)	335 (23.5)
Both DVT and PE	226 (8.9)	240 (9.4)	167 (11.7)	168 (11.8)
Neither DVT nor PE^c^	3 (0.1)	3 (0.1)	1 (0.1)	1 (0.1)

*DVT* deep vein thrombosis, *PE* pulmonary embolism, *SD* standard deviation

^a^REMEDY included 567 patients in the dabigatran group, and 606 patients in the warfarin group, that rolled over from RE-COVER and RE-COVER II

^b^Results of objective testing for initial symptomatic DVT/PE performed locally. If a patient had more than one event, the last event prior to randomization was classified as the qualifying event

^c^These were diagnosed with DVT or PE initially but refuted on subsequent local examination

### Net clinical benefit

**Fig. 1:**
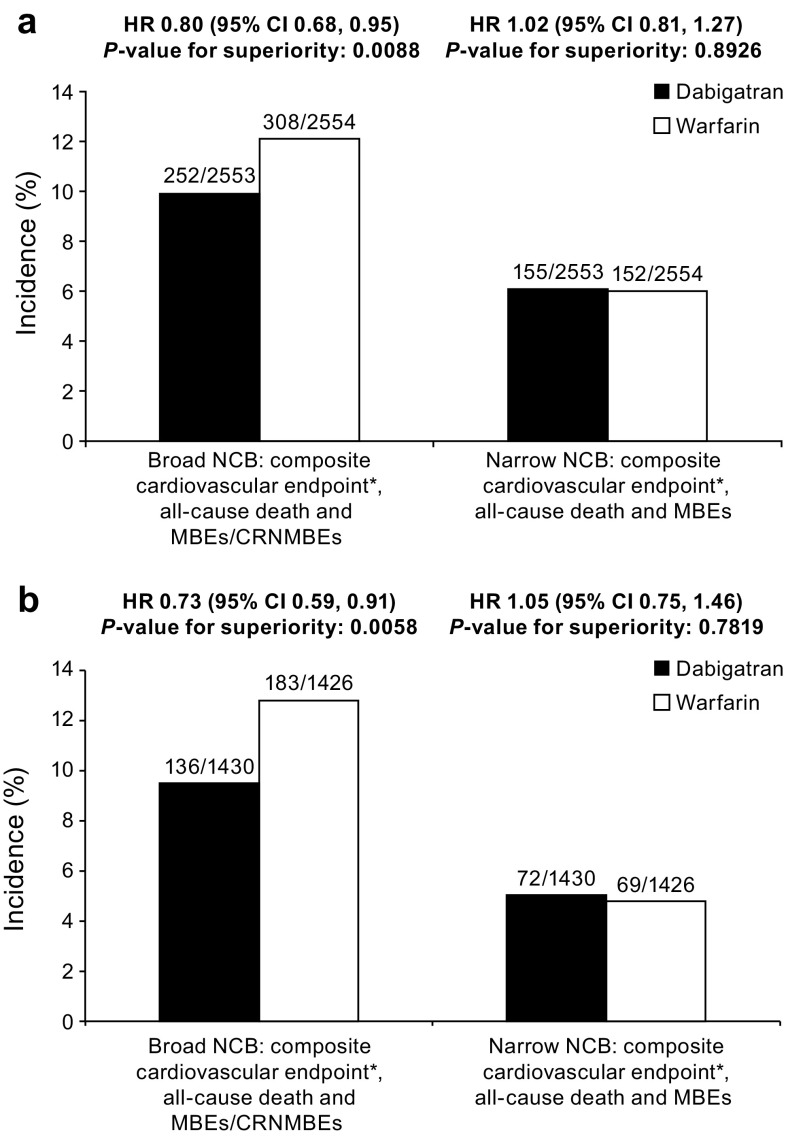
Net clinical benefit for dabigatran vs. warfarin in **a** RE-COVER/RE-COVER II pooled data and **b** RE-MEDY. *Non-fatal events of recurrent VTE, MI, stroke, or systemic embolism. *CI* confidence interval, *CRNMBE* clinically relevant non-major bleeding event, *HR* hazard ratio, *MBE* major bleeding event, *MI* myocardial infarction, *NCB* net clinical benefit, *VTE* venous thromboembolism

When NCB was defined to include MBEs as the only bleeding outcome (narrow definition), NCB was similar between DE and warfarin (RE-COVER/RE-COVER II, HR 1.02; 95% CI 0.81–1.27 and RE-MEDY, HR 1.05; 95% CI 0.75–1.46) (Fig. 1a, b).

When MBEs plus CRNMBEs were included as bleeding outcomes (broad definition), NCB was superior with DE compared with warfarin [RE-COVER/RE-COVER II, hazard ratio (HR) 0.80; 95% CI 0.68–0.95 and RE-MEDY, HR 0.73; 95% CI 0.59–0.91] (Fig. 1a, b).

In subgroups divided according to cTTR, the NCB (both definitions) with DE was similar to warfarin, regardless of warfarin cTTR in both the RE-COVER/RE-COVER II and the RE-MEDY analyses, with no trends observed, whether CRNMBEs were included as bleeding outcomes or not. This result was observed when centers were grouped into quintiles (Tables 2, 3) and when they were grouped into tertiles (data not shown), according to their mean TTR (INR 2–3) and overall number of patients. As only centers with ≥1 patient with available TTR are included, these cTTR data are limited to 5055 patients vs. the 5107 patients in the study overall for the RE-COVER/RE-COVER II analysis, and 2813 patients vs. the 2856 patients in the study overall for the RE-MEDY analysis.

**Table 2:** Event rates for the composite cardiovascular endpoint including MBE and all death, with or without CRNMBE, stratified by center TTR in RE-COVER/RE-COVER II

Center TTR category^a^	Including CRNMBE*		Excluding CRNMBE**	
	Dabigatran	Warfarin	Dabigatran	Warfarin
<47.1				
Patients, N	516	531	516	531
Event rate, %	11.0	16.2	8.3	9.0
HR vs. warfarin (95% CI)	0.66 (0.47, 0.92)		0.92 (0.61, 1.39)	
47.1 to <57.7				
Patients, N	450	489	450	489
Event rate, %	8.4	9.6	5.3	4.3
HR vs. warfarin (95% CI)	0.86 (0.56, 1.31)		1.24 (0.69, 2.22)	
57.7 to <61.9				
Patients, N	555	530	555	530
Event rate, %	8.5	10.9	4.1	5.8
HR vs. warfarin (95% CI)	0.76 (0.52, 1.12)		0.71 (0.41, 1.21)	
61.9 to <68.0				
Patients, N	481	492	481	492
Event rate, %	9.4	13.4	5.8	5.5
HR vs. warfarin (95% CI)	0.67 (0.46, 0.98)		1.05 (0.62, 1.78)	
≥68.0				
Patients, N	501	510	501	510
Event rate, %	12.6	10.0	7.0	4.9
HR vs. warfarin (95% CI)	1.27 (0.88, 1.84)		1.43 (0.86, 2.39)	

HR obtained from Cox Model with treatment, center TTR and treatment by center TTR interaction stratified by study

*CI* confidence interval, *CRNMBE* clinically relevant non-major bleeding event, *HR* hazard ratio, *INR* international normalized ratio, *TTR* time in therapeutic range

*P value for treatment by center TTR interaction: 0.0815

**P value for treatment by center TTR interaction: 0.3896

^a^Centers grouped into five categories according to their mean TTR (INR 2–3) and overall number of patients (quintiles). Only centers with at least one patient with available TTR (INR 2–3) were included

**Table 3:** Event rates for the composite cardiovascular endpoint including MBE and all death, with or without CRNMBE, stratified by center TTR in RE-MEDY

Center TTR category^a^	Including CRNMBE*		Excluding CRNMBE**	
	Dabigatran	Warfarin	Dabigatran	Warfarin
<49.3				
Patients, N	264	298	264	298
Event rate, %	8.0	13.1	4.2	7.4
HR vs. warfarin (95% CI)	0.58 (0.34, 0.99)		0.54 (0.26, 1.12)	
49.3 to <59.3				
Patients, N	284	291	284	291
Event rate, %	7.4	12.0	4.9	3.4
HR vs. warfarin (95% CI)	0.58 (0.34, 1.00)		1.42 (0.63, 3.19)	
59.3 to <67.0				
Patients, N	268	288	268	
Event rate, %	10.8	13.5	5.6	
HR vs. warfarin (95% CI)	0.76 (0.47, 1.23)		1.04 (0.51, 2.12)	
67.0 to <73.1				
Patients, N	283	276	283	276
Event rate, %	11.3	12.3	4.9	4.3
HR vs. warfarin (95% CI)	0.93 (0.57, 1.51)		1.16 (0.54, 2.51)	
≥73.1				
Patients, N	289	272	289	272
Event rate, %	10.0	13.2	5.2	3.7
HR vs. warfarin (95% CI)	0.78 (0.48, 1.26)		1.48 (0.66, 3.28)	

HR obtained from Cox Model with treatment, center TTR, and treatment by center TTR interaction

*CI* confidence interval, *CRNMBE* clinically relevant non-major bleeding events, *HR* hazard ratio, *INR* international normalized ratio, MBE major bleeding event, *TTR* time in therapeutic range

*P value for treatment by center TTR interaction: 0.6545

**P value for treatment by center TTR interaction: 0.3508

^a^Centers grouped into five categories according to their mean TTR (INR 2–3) and overall number of patients (quintiles). Only centers with at least one patient with available TTR (INR 2–3) were included

## Discussion

Phase 3 trials have shown DE to be as effective as warfarin for the treatment of acute VTE and for the extended treatment of VTE, with a lower risk of bleeding [5, 6, 8]. Whereas clinical trials tend to treat benefits and risks as separate entities, evaluation of the NCB can provide a clearer representation of the benefit–risk balance of a treatment overall by analyzing efficacy and safety as a collective outcome.

The inclusion of CRNMBEs in the evaluation of NCB provides a comprehensive reflection of anticoagulant safety outcomes encountered in real-world clinical practice [9]. This is because CRNMBEs, which include bleeding leading to hospitalization or requiring surgical treatment, could adversely affect prognosis and can also result in reduced patient adherence to, and persistence with, necessary anticoagulant therapy [12, 13].

Although NCBs of DE and warfarin were similar when the NCB included only MBEs as the bleeding outcome, the NCB of DE was superior to that of warfarin when CRNMBEs were also included in the calculation.

It was surprising that the analysis of NCB stratified by cTTR showed that quality of warfarin control did not influence the relative benefits of dabigatran and warfarin for the treatment and secondary prevention of VTE. This was true when either the broad or the narrow NCB definitions were used.

### Study strengths and limitations

RE-COVER, RE-COVER II and RE-MEDY were randomized, double-blind studies with central adjudication of outcome events. RE-MEDY is the only study so far of a DOAC with warfarin as the comparator in the extended treatment of VTE. The NCB definitions included clinically relevant cardiovascular endpoints (including stroke and systemic embolism) and all-cause mortality, as well as bleeding.

One limitation is that the endpoints included in the NCB definition do not have an equal impact on morbidity and mortality, but were weighted equally in this analysis. Furthermore, in analyses on the association of clinical effects of DE with quality of warfarin control (cTTR), limited data were presented, as these were dependent on the availability of patient INR measurements. Finally, this was a post-hoc analysis.

These analyses of safety and efficacy data support previous assessments of the benefit–risk balance of DE vs. warfarin [5, 6, 8].

## Conclusion

The NCB of DE is superior to that of warfarin when the NCB definition includes MBEs plus CRNMBEs (typical of the safety outcomes arising in real-world clinical practice). This applies to both the initial treatment and the extended treatment of VTE. The NCB of DE is similar to warfarin when NCB includes only MBEs as the bleeding outcome.

These results indicate a positive impact of DE, in comparison with warfarin, on the clinical outcome of patients treated for acute VTE or for secondary VTE prevention in clinical practice settings, regardless of the quality of INR control.
